# Assessment of resistance to colicinogenic synthetic phage antimicrobial system

**DOI:** 10.1128/spectrum.00793-24

**Published:** 2024-10-15

**Authors:** Meghan McGillin, Jeffrey I. Tokman, Ella Hsu, Samuel D. Alcaine

**Affiliations:** 1 Department of Food Science, Cornell University, Ithaca, New York, USA; The University of Tennessee Knoxville, Knoxville, Tennessee, USA

**Keywords:** phage therapy, phage resistance, persistence, bacteriocins, tolerance

## Abstract

This work presents a multi-hurdle approach that addresses antimicrobial resistance by minimizing the selective pressure of antimicrobials using a novel colicinogenic–phage system. We have created two synthetic T7 phages (T7-E1 and T7-M) by inserting the gene of colicin E1 (Cea) or colicin M (Cma) into the genome of the T7 phage, thereby adding an additional colicin-based hurdle to the T7 lytic cycle. The colicin–phages' efficacy in suppressing the outgrowth of a T7-resistant sub-population within a mixed culture of *Escherichia coli* was demonstrated using a challenge matrix design under planktonic and structured conditions. When T7-resistant cells were present at 1% of the total planktonic population, T7-E1 delayed the outgrowth. At 0.1% resistance, T7-M delayed resistant outgrowth, whereas T7-E1 suppressed the resistant sub-population. When T7-E1 and T7-M were combined into a triple-hurdle treatment, the T7-E1/T7-M cocktail completely suppressed a mixed planktonic population of 50% resistance cell concentrations. In structured environments, the colicin–phage treatments formed clear and confluent plaque-like zones of clearing in the mixed populations of 50% resistant cells with a lawn density of 1 × 10^6^ CFU/mL. Reducing the lawn density to 1 × 10^5^ CFU/mL diminished the multi-hurdle treatments' effectiveness, as demonstrated by localized zones of clearing within turbid bacterial lawns, highlighting the relationship between bacterial lawn density and phage effectiveness in structured environments. Fluctuation assays revealed persistence as the predominant mechanism for overcoming the treatments by T7-sensitive *E. coli*. Results indicate that T7-M treatment significantly reduces persister formation compared to WT-T7, while T7-E1 unexpectedly increases persister formation significantly. This suggests a complex relationship between antimicrobial stress and persister formation.

**IMPORTANCE:**

Antimicrobial resistance (AMR) poses a significant challenge in treating bacterial infections. To address this, we present a multi-hurdle approach that combines the power of different antimicrobials to target resistance. We have weaponized the natural predator of *Escherichia coli*, the T7-phage, by engineering it to produce toxins called colicins, resulting in a colicin–phage antimicrobial. This multi-hurdled approach aims to decrease resistance risk because survival requires different tactics to overcome the phage and colicin activity, thus adding a hurdle in a bacterium's pathway to resistance. In cases of pre-existing resistance, the colicin effectively controlled the sub-population resistant to the phage. When investigating the emergence of resistance, we discovered that antimicrobial persistence was the predominant survival strategy. These findings reveal an essential slice of the AMR pie by emphasizing bacterial survival tactics that are not based on resistance genes. By expanding our AMR lens to include persistence, we can more effectively address treatment failure.

## INTRODUCTION

The rise of multi-drug resistance (MDR) pathogenic bacteria is an increasing global problem. Organizations like the World Health Organization and the US Centers for Disease Control have issued warnings about the threat to public health that these MDR pathogens represent and the need for alternative antimicrobials (1).

One such treatment is bacteriophage (phage) therapy (2–4). Phages are viruses that infect bacteria for the purpose of replicating. Their infection cycle concludes with a fatal lysis of the host, which releases the phage progeny to perpetuate the infection cycle (5). The replicative nature of phage predation provides an amplified effect (auto-dosing) that distinguishes it from small-molecule therapeutics (5). This, coupled with their specificity, enables targeting inhibition with minimal disruption to the overall microbiota positions phage therapy as a compelling alternative to traditional broad-spectrum antibiotics (6).

Resistance is an unavoidable challenge for all antimicrobials, and bacteria have evolved numerous anti-phage mechanisms to evade infection (5). A phage cocktail containing multiple strains is one approach to discourage the target organism from developing resistance to the treatment (7). However, *Escherichia coli* strains resistant to one phage are more likely to be resistant or develop resistance to other phages. Similarly, bacteria resistant to one type of antibiotic are likely to be resistant to others as the mechanisms that impart phage resistance can be quite similar (8–12). This necessitates the development of novel treatment methods that can prevent or delay the establishment of bacterial resistance to phages (12).

Advances in synthetic biology have enabled the augmentation of natural phage properties to overcome these limitations in phage therapy. Previous studies have engineered T7 to express various genes that enhance bacterial killing. For instance, phages have been engineered to deliver depolymerases to degrade exopolysaccharide capsules and biofilms, express quorum-quenching enzymes, and expand host range through a tail exchange strategy (13–16). These studies highlight the potential of synthetic biology to enhance phage therapy and address broader questions relevant to the present study.

Our proof-of-principle colicinogenic–phage system leverages synthetic biology by integrating colicin co-expression within the T7-phage infection cycle, producing a multi-hurdle approach to target resistant *E. coli* (17, 18). Colicins are a class of bacteriocins specifically produced by *E. coli* that inhibit the growth or kill other strains of *E. coli*. Colicins were chosen because, like phages, they are more specific in their targets than broad-spectrum antibiotics, thus having a smaller impact on commensal bacteria. Colicin M and E1 mechanisms both affect the cell membrane (19). Colicin M binds to murein precursors, preventing peptidoglycan biosynthesis, resulting in autolysis of the cell, whereas colicin E1 is a channel-forming peptide (20–23).

**Fig 1:**
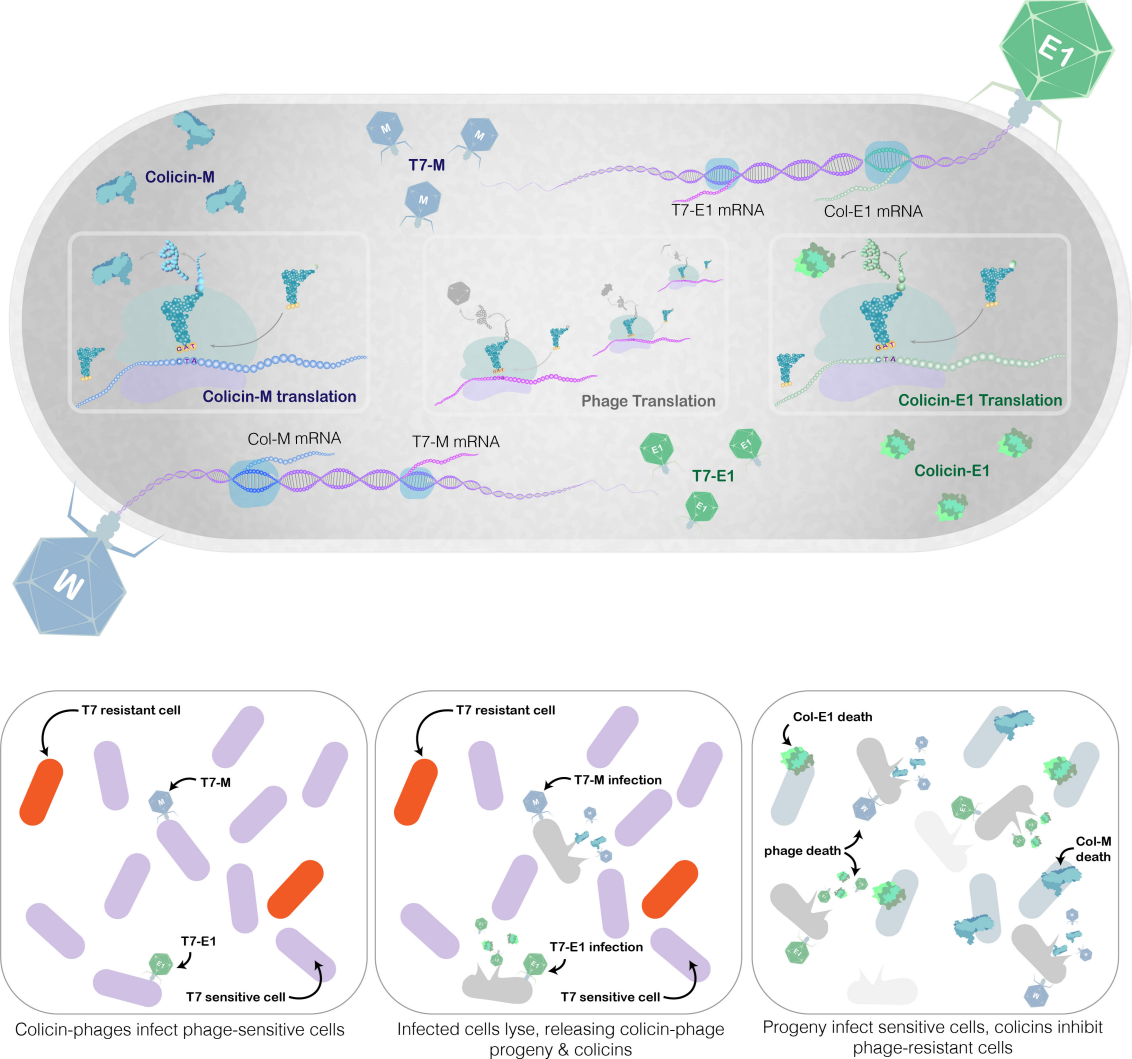
Graphic abstract illustrating lytic infection cycle of modified colicin–phage. Injection of modified T7 genome containing colicin gene (*cea* for colicin E1 or *cma* for colicin M) following absorption results in co-expression of the colicin phage and colicins, which are released during host lysis.

**Fig 2:**
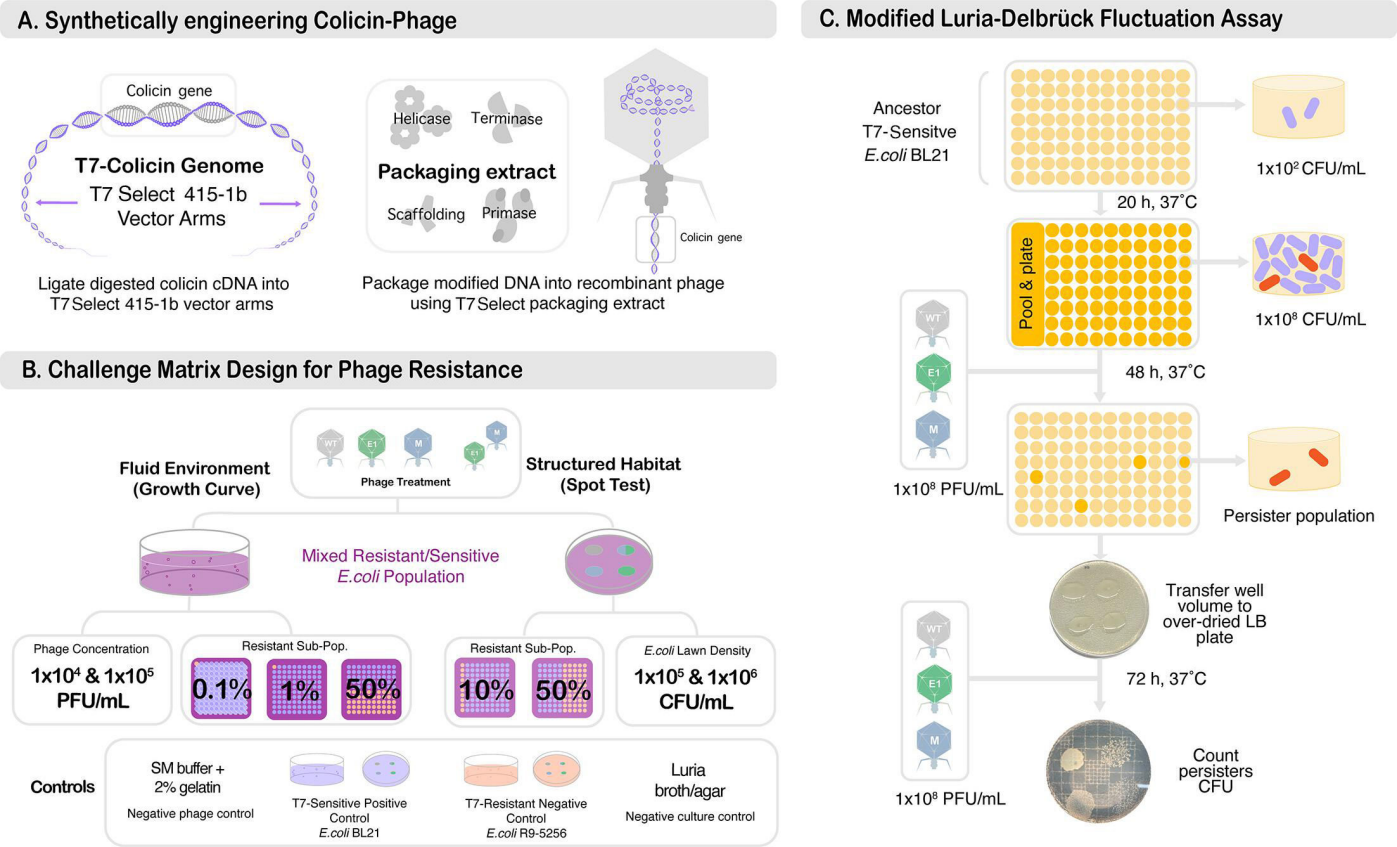
Experiment overview. (**A**) Synthetic engineering of the colicin–phage treatments using the T7Select 415-b kit. (**B**) Challenge matrix design overview for structured and unstructured environments. (**C**) Luria–Delbrück fluctuation assay modified for persister frequency as described by Hossain et al. (2023).

The novelty in this multi-hurdled approach is T7’s role as an antimicrobial agent and indirectly as an antimicrobial generator (Fig. 1). The multi-hurdle system was constructed by inserting the gene of an *E. coli* targeting Colicin E1 (Cea) or Colicin M (Cma) into the genome of the T7 phage, resulting in two synthetic T7 phages (T7-E1 and T7-M, respectively) capable of co-expressing a colicin, thereby, adding a secondary colicin-based hurdle to the T7 lytic cycle (Fig. 2A).

The overarching goal of this work is to understand the impact of additional hurdles in (i) inhibiting phage resistance within a mixed population of phage susceptibilities and (ii) characterizing the survival mechanism the multi-hurdle system selects for within an entirely sensitive population (Fig. 2B and C). By doing so, we hope to shed more light on a combinational treatment’s potential to reduce the burden of treatment failure.

Acknowledging the influence of the environment on phage-host dynamics, we employed a challenge matrix design. In this design, different bacterial populations (*E. coli*) varying by prevalence of resistance were challenged by different phage treatments.

**Fig 3:**
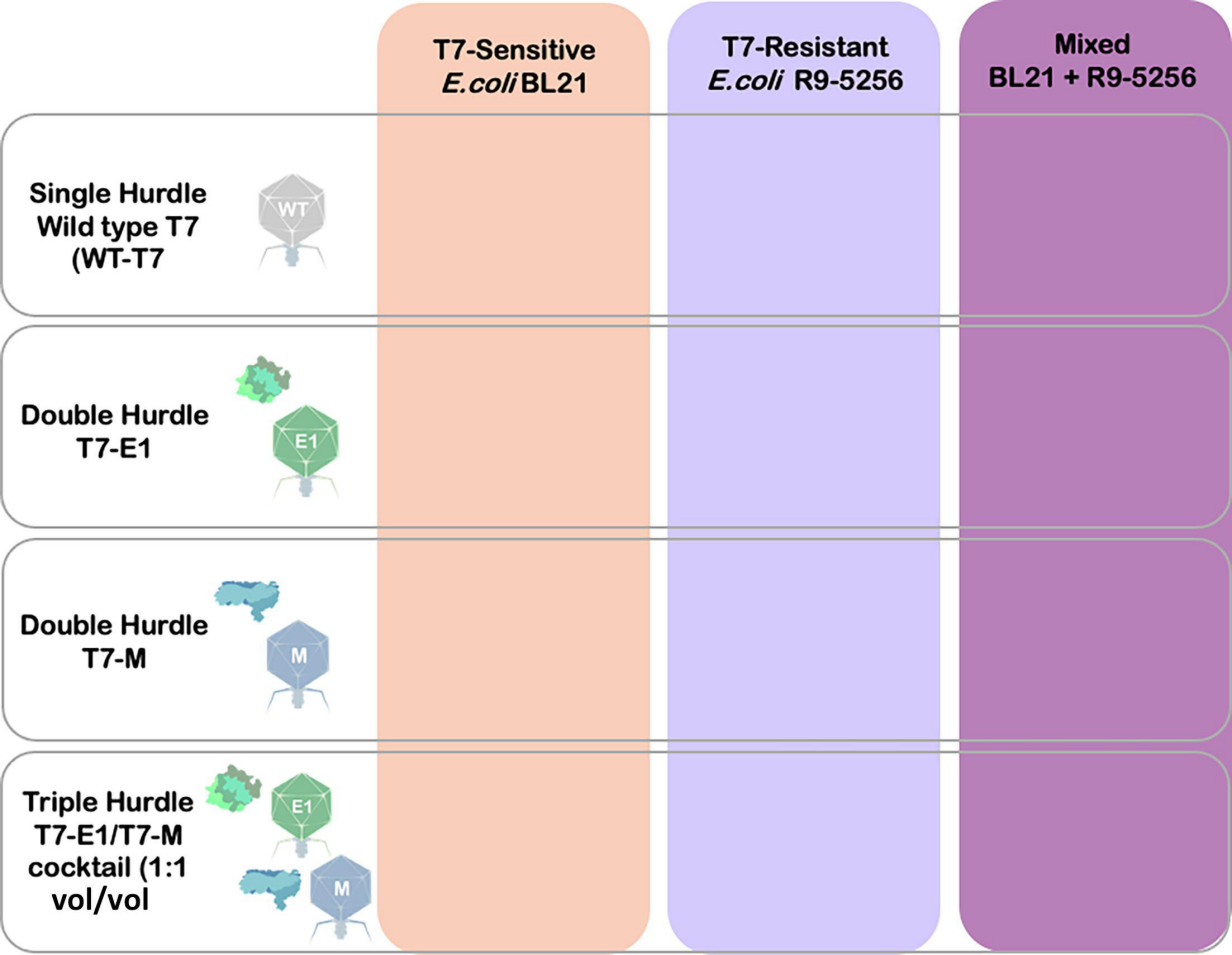
Challenge matrix structure.

We employed a matrix structure to systemically challenge different phage treatments against bacterial populations of varying resistance ratios to evaluate the effects of multi-hurdle approaches under both structured and non-structured habitats (Fig. 3) (24). Under structured conditions resembling biofilms and host tissue, we found that the lawn density of the mixed population influenced the efficacy of the colicin–phage treatments more than the treatment’s number of hurdles or prevalence of resistance within the mixed population (25). Under planktonic conditions, which are associated with early stages of infection and host dissemination, we found a relationship between the treatment’s number of hurdles and the prevalence of resistance, influencing whether the treatment fully suppressed or delayed mixed population outgrowth (26).

We used a modified fluctuation assay to better understand how sensitive bacteria overcome the cumulative stressors of multi-hurdle treatments (27). Our results show that the predominant survival strategy is antimicrobial persistence arising from population-level phenotypic heterogeneity as opposed to spontaneous mutation-induced canonical resistance (28). Moreover, we report that the mode of action by the second hurdle may influence persister formation, and in some cases, the combined stress of the phage and colicin can induce persister formation at a greater likelihood than when applied individually. Collectively, this work underscores the nuance of antimicrobial resistance, highlighting the versatility of non-canonical resistance and challenging the notion that more is better in terms of treatment outcome.

## MATERIALS AND METHODS

### Phages and bacterial strains

*E. coli* BL21 and bacteriophage T7Select 415–1 were purchased from EMD Millipore (Billerica, Massachusetts). Wild-type bacteriophage T7 was gifted from Dr. Sam Nugen. The pathogenic *E. coli* O104:H4 strain, FSL R9-5256, was given for use by Dr. Martin Wiedmann (USDA, Pina Fratamico via Martin). Overnight cultures of *E. coli* strains used in this study were grown at 37°C with shaking at 220 rpm in 5 mL of Luria Broth (LB), pH 7.5, in 16-mm culture tubes. Bacterial lawns were prepared using a double agar overlay method on LB to determine the titer of phage samples.

### Construction of engineered bacteriophage

We modified bacteriophage T7 to carry the *E. coli* gene encoding colicin E1 (Cea) or colicin m (Cma) (Fig. 2A). We designed constructs (Supplemental S1–3) of 1,702 and 949 base pairs for *cea* and *cma,* respectively, containing the EcoRI restriction digest site, T7 promoter site, T7 RNA polymerase binding site, *cea* or *cma*, His-tag, and the HindIII restriction digest site, which were constructed within a PCC1 cloning vector by Genscript USA Inc. (Piscataway, NJ). The *cea* and *cma* constructs were amplified using M13 forward and reverse primers with Phusion High-Fidelity DNA Polymerase (New England Biolabs, Ipswich, MA) before being purified using the QIAquick PCR Purification Kit (Qiagen, Valencia, CA). After purification, the constructs were digested using EcoRI and HindIII restriction enzymes from New England Biolabs (Ipswich, MA). The respective construct was then ligated into the T7Select 415–1 genome vector arms using T4 DNA ligase (New England Biolabs, Ipswich, MA), resulting in their insertion within the multiple cloning sites downstream of the 10B protein. Finally, the modified genome was packaged using T7Select packing kits to create T7-E1 and T7-M.

The phages were then plated on lawns containing BL21 after packaging. Individual plaques were selected, and a PCR screen was done using T7Select Up and Down primers to look for inserts of the correct size for their respective colicin constructs, and plaques containing inserts of the correct size were propagated on *E. coli* BL21. The propagated phages were filtered using 0.22-µm SFCA filters (Corning Life Sciences, Corning, NY) before centrifugation at 20,000 *g* for 1.5 h. After centrifugation, the supernatants were saved for further testing. The remaining phage pellets were resuspended by adding SM Buffer with gelatin (Cold Spring Harbor) and let to sit overnight. Samples were centrifuged at 20,000 *g* for 1.5 h for a second time, and the supernatant was discarded to reduce background colicin levels present from the initial propagation. Fresh SM Buffer with gelatin was added to the phage pellets and allowed to sit overnight at 4°C and kept as T7-E1 and T7-M phage stocks for further experiments.

### Confirmation of colicin production

To determine that the colicin proteins were indeed being produced after phage infection on *E. coli* BL21, we precipitated the proteins from the supernatant of the modified phages using acetone precipitation (29). The lysates were resuspended in 2× Laemmli buffer and separated using gel electrophoresis on a precast Mini-PROTEAN TGX gel (Any Kd; Bio-Rad, Hercules, CA). The gels were transferred to a PVDF membrane using the Trans-Blot Turbo Transfer System (Bio-Rad, Hercules, CA). Membranes were incubated in blocking reagent {TTBS [Tween–5% (wt/vol), PBS–0.1% (vol/vol)] +skim milk powered–5% [wt/vol]} for 2 h with mouse anti-his antibody (1/500) at room temperature (Thermo Fisher Scientific, Waltham, MA). Blots were rinsed five times for 5 min in TTSB and incubated with Goat anti-mouse IGG AFP 488 antibody (1/3,000) overnight at room temperature (Thermo Fisher Scientific, Waltham, MA). The blots were analyzed using Image Lab software (Bio-Rad, Hercules, CA) (supplemental S4).

### Determination of a T7-resistant strain and colicin-sensitive strains

To identify T7-resistant and colicin-sensitive *E. coli* strains, an internal library of *E. coli* strains was screened using the double agar overlay method to create individual lawns of each strain. Each lawn population was challenged with 5 µL of the supernatant from a stock of wild-type T7, T7-E1, and T7-M phage. The plates were incubated for 24 h at 37°C. Plates that did not form plaques with the wild-type T7 but showed clearings with the colicin–phage supernatant were used to select strains that were resistant to wild-type T7 and susceptible to either of the colicins.

### Challenge matrix in structured and planktonic environments

Acknowledging the influence of the environment on phage-host dynamics, we employed a challenge matrix design. In this context, "matrix" refers to a structured format where different variables, such as phage treatment and bacterial population susceptibility, are systematically arranged (Fig. 3). Using this design, we evaluated the inhibitory effects of single-, double-, and triple-hurdled phage treatments against T7-sensitive (*E. coli* BL21), T7-resistant (*E. coli* R9-5256), and mixed resistant/sensitive (*E. coli* BL21 and *E. coli* R9-5256) populations (Fig. 2B). These evaluations were conducted under both structured and non-structured habitats.

#### Structured environment challenge matrix

Agar gel was used as a simplified model for a spatially structured habitat of seven bacterial populations distinguished by T7-resistance and lawn density (Table 1). A single colony of *E. coli* BL21 and *E. coli* R9-556 were each inoculated in 5 mL of LB broth at 37 ˚C with shaking until it reached an OD_600_ of ~0.7 nm. The overnight cultures were each diluted in LB to achieve a final plate concentration of 1 × 10^5^ and 1 × 10^6^ CFU/mL (Table 1).

**TABLE 1:** Spot assay population matrix parameters

Phage resistance	Culture population	Plate concentration
0% (sensitive)	*E. coli* BL21	1 × 10^5^ CFU/mL
0% (sensitive)	*E. coli* BL21	1 × 10^6^ CFU/mL
10% (mixed)	*E. coli* BL21	1 × 10^6^ CFU/mL
	*E. coli* R9-5256	1 × 10^5^ CFU/mL
50% (mixed)	*E. coli* BL21	1 × 10^5^ CFU/mL
	*E. coli* R9-5256	1 × 10^5^ CFU/mL
50% (mixed)	*E. coli* BL21	1 × 10^6^ CFU/mL
	*E. coli* R9-5256	1 × 10^6^ CFU/mL
100% (resistant)	*E. coli* R9-5256	1 × 10^5^ CFU/mL
100% (resistant)	*E. coli* R9-5256	1 × 10^6^ CFU/mL

For the sensitive and resistant populations, 300 µL of the appropriate diluted cultures were inoculated in 3 mL of LB top agar (7.5% agarose), vortexed briefly, and poured over LB agar. For the 10% and 50% mixed population, 150 µL of each culture from the appropriate culture dilution were added to the top-agar prior to plating (Table 1).

Two microliters of each phage treatment was spotted on the surface of the plates at a titer of 1 × 10^7^ PFU/mL. The plates were incubated at room temperature for 48 h, followed by inspection of lysis zones. Each treatment was done in technical duplicates. Biological triplicates of the assays were performed on separate days.

#### Unstructured (liquid) environment challenge matrix

A growth curve-based challenge matrix was employed to evaluate treatment effectiveness in fluid habitats containing populations of varying T7-susceptibilities. In total, six assays were conducted to determine the effect of phage treatment at 1 × 10^4^ and 1 × 10^5^ PFU/mL against the outgrowth of the mixed cell population containing varying levels of resistance under planktonic conditions (Table 2).

**TABLE 2:** Planktonic environment challenge matrix factors^*a*^

Phage resistance level (%)	Resistant sub-population concentration (CFU/mL)	Phage concentration (PFU/mL)
0% (sensitive)	0	1 × 10^4^
0% (sensitive)	0	1 × 10^5^
0.1% (mixed)	1 × 10^4^	1 × 10^4^
0.1% (mixed)	1 × 10^4^	1 × 10^5^
1% (mixed)	1 × 10^5^	1 × 10^4^
1% (mixed)	1 × 10^5^	1 × 10^5^
50% (mixed)	1 × 10^7^	1 × 10^4^
50% (mixed)	1 × 10^7^	1 × 10^5^
100% (resistant)	1 × 10^7^	1 × 10^4^
100% (resistant)	1 × 10^7^	1 × 10^5^

^*a*^ The starting inoculum level for the sensitive sub-population remained constant at 1 × 10^7^ CFU/mL in the mixed and sensitive populations.

**Fig 4:**
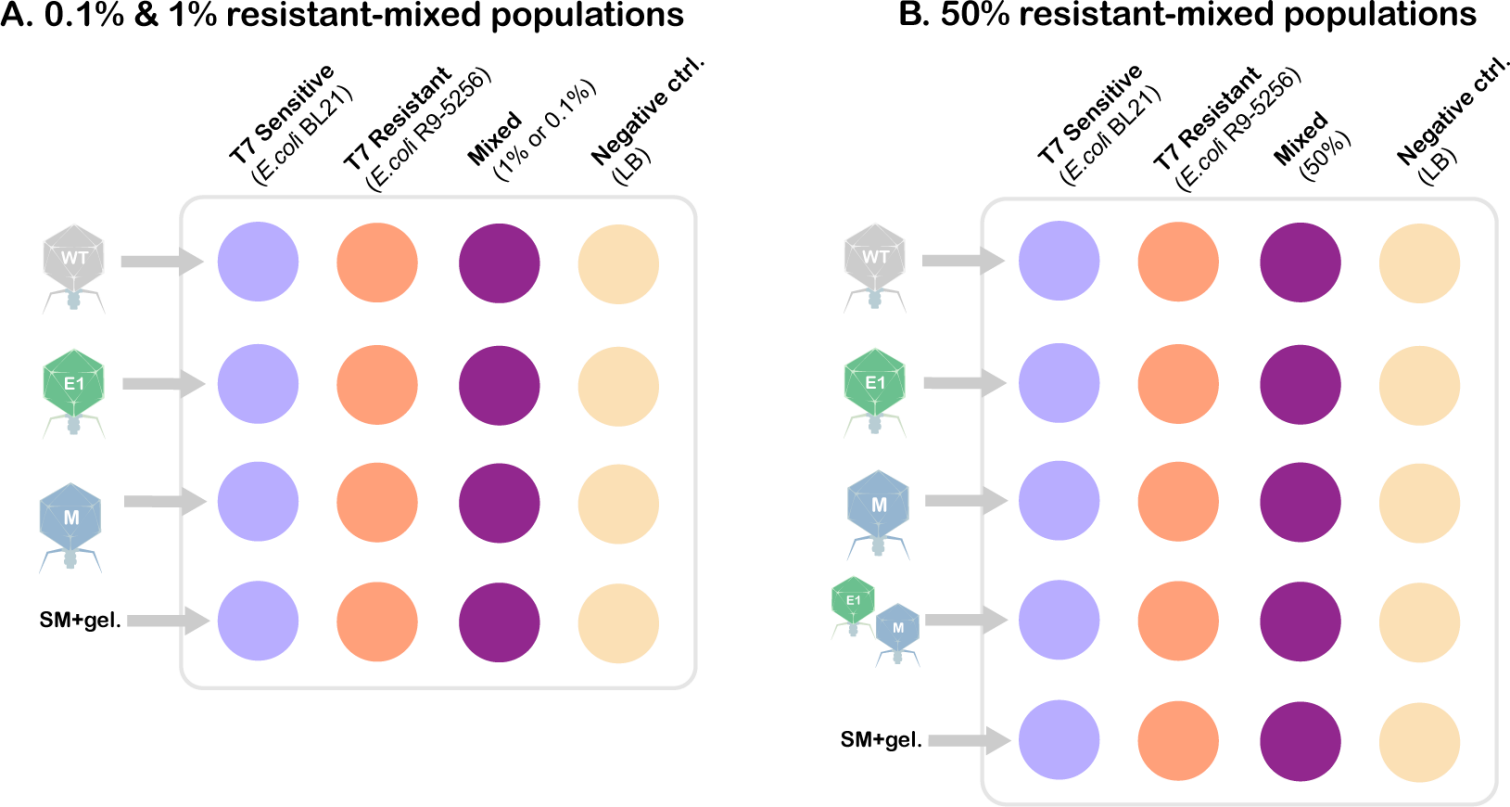
Well-plate matrix design for unstructured planktonic habitat. (**A**) 1% and 0.1% resistance assay. (**B**) 50% resistance assay with additional cocktail treatment. Each assay was challenged with the appropriate phage treatment at a concentration of 1 × 10^4^ and 1 × 10^5^ PFU/mL.

Figure 4 illustrates the composition of the challenge matrix. Briefly, a single colony each of T7-sensitive *E. coli* BL21 and T7-resistant *E. coli* R9-5256 were each inoculated in 5 mL of LB broth at 37 ˚C with shaking until it reached an OD_600_ of ~0.7 nm. Overnight cultures of *E. coli* BL21 and *E. coli* FSL R9-5256 were diluted to a concentration of 1 × 10^7^ CFU/mL before transferring to their designated wells in a 24-well plate (sensitive and resistant, respectively) (VWR, Radnor, PA).

The mixed populations were cocultured 1:1 (vol/vol) and transferred to their designated well. The prevalence of resistance within the mixed population varied across assays. Within the mixed populations, T7-sensitive *E. coli* BL21 (1 × 10^7^ CFU/mL) was cocultured with T7-resistant *E. coli* R9-5256 under the following concentrations: (i) 1 × 10^7^ CFU/mL of *E. coli* R9-5256 for the 50% ratio, (ii) 1 × 10^5^ CFU/mL for the 1% ratio, and (iii) 1 × 10^4^ CFU/mL for the 0.1% ratio (Table 2). Each well had a final culture volume of 1 mL (Table 2).

Each bacterial population was exposed to the following treatments at 1 × 10^4^ and 1 × 10^5^ PFU/mL: wild-type T7 (WT-T7), T7-E1, T7-M, and SM +2% gelatin (negative treatment control) (Fig. 4A). For the assay containing the 50% mixed population, a T7-E1/T7-M cocktail (1:1 vol/vol) was included as an additional treatment (Fig. 4B).

For each assay, the 24-well plate was sealed with sterile film (Thermo Fisher Scientific, Waltham, MA) before being incubated at 37°C with shaking at 200 rpm for 24 h. Wells were monitored for optical density at 600 nm (OD_600_) every 5 min using a BioTek Synergy H1 hybrid reader. All assays were performed in biological triplicate on separate days.

### Persistence frequency fluctuation assay

The experimental procedures employed in this study were adapted based on the methodology established by Hossain et al. (Fig. 2C) (27). Briefly, overnight cultures of *E. coli* BL21 were diluted to ~1 × 10^2^ CFU/mL in M9LB. To initiate a sizable number of parallel isogenic cultures, 190-µL aliquots were transferred to 96 wells (96 technical replicates) of a 96-well plate (VWR, Radnor, PA).

After 20 h of incubation at 37 ˚C with 200 rpm, 16 of the isogenic cultures were pooled before plating on LB agar to determine the average number of cells prior to treatment. The remaining 80 cultures were inoculated with 10 µL of the appropriate phage (~1 × 10^8^ PFU/well) for a final well volume of 200 µL and inoculated at 37 ˚C with 200 rpm for 48 h. Prior to enumeration, the isogenic cultures were re-inoculated with 10 µL of the appropriate phage treatment (~1 × 10^8^ PFU/well) to eliminate false-positive persister survival. After reinoculation, the cultures were transferred onto overdried plates to enumerate persistence (30). Plates were incubated at 37 ˚C for 72 h, and persister colony-forming units (CFU) were counted. Persister frequency was determined by dividing the mean CFU of surviving cells by the original CFU of suspension (27, 31). All assays were repeated in biological triplicates on separate days.

### Descriptive statistics

All analyses were performed using R statistical software version 3.5.0 (32). We utilized a negative binomial mixed-effects model to analyze the relationship between persister CFU and phage treatments while accounting for trial-specific variability using the glmer.nb function (33). The model included a fixed effect for treatment and a random intercept for each trial.

We conducted *post-hoc* comparisons of T7-E1 and T7-M against WT using estimated marginal means (EMM) using the dunnettx method for a *P*-value correction to investigate specific differences between treatment type after the initial negative binomial mixed-effects model analysis using the emmeans package v1.3.5 (34). Comparing EMMs facilitated the assessment of the impact of the colicin-hurdle treatment on persister CFUs in comparison to the WT-T7 treatment. Persister frequency data were visualized using ggplot and ggbreaks (35, 36).

## RESULTS AND DISCUSSION

### Challenge matrix

#### Structured environment

It is well established that the dynamics between phages and hosts are influenced by the surrounding environment (24). Structured habitats introduce factors, such as increased viscosity, which can impede phage diffusion and subsequently immobilize cells, potentially reducing contact with their host. Considering the spatial structure present in environments such as biofilms, soils, sediments, and host tissues, it is crucial to examine phage-host dynamics in conditions where bacteria are not freely mixing (25). Using an agar gel as a simplified model for a spatially structured habitat, we developed a challenge matrix spot test to evaluate the impact of multi-hurdled phage treatments against bacterial populations of varying T7-susceptibilities (Table 1) in a more viscous environment.

**Fig 5:**
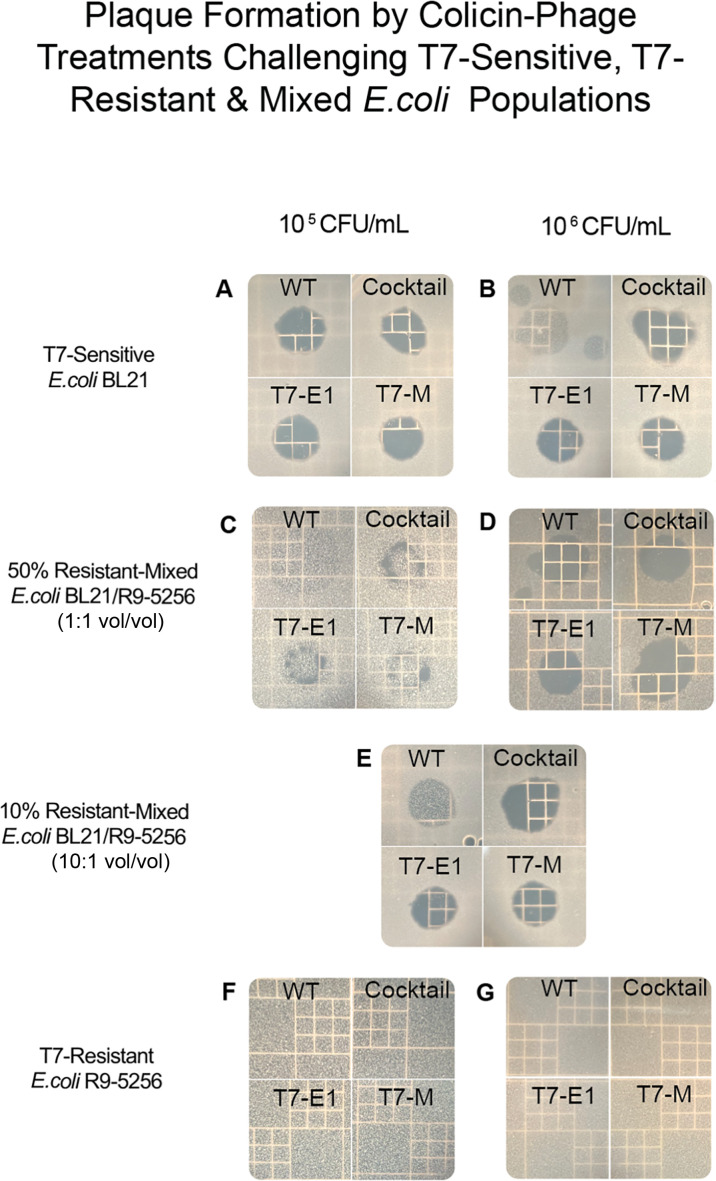
Structured challenge matrix plates containing T7-sensitive, T7-resistant, and mixed bacterial lawns challenged with T7-WT, T7-E1, T7-M, and T7-E1/T7-M cocktail (1:1 vol/vol). (**A**) T7-sensitive cells (*E. coli* BL21) at 1 × 10^5^ CFU/mL challenged by phage population at 1 × 10^7^ PFU/mL. (**B**) T7-sensitive cells (*E. coli* BL21) at 1 × 10^6^ CFU/mL challenged by phage population at 1 × 10^7^ PFU/mL. (**C**) 50% T7-resistant-mixed population (1:1 vol/vol *E. coli* BL21 and *E. coli* R9-5256) at 1 × 10^5^ CFU/mL challenged by phage population at 1 × 10^7^ PFU/mL. (**D**) 50% T7-resistant-mixed population (1:1 v/v *E. coli* BL21 and *E. coli* R9-5256) at 1 × 10^6^ CFU/ml challenged by phage population at 1 × 10^7^ PFU/mL. (**E**) 10% T7-resistant-mixed population (*E. coli* BL21 at 1 × 10^6^ CFU/mL and *E. coli* R9-5256 at 1 × 10^5^ CFU/mL) challenged by phage population at 1 × 10^7^ PFU/mL. (**F**) T7-resistant cells (*E. coli* R9-5256) at 1 × 10^5^ CFU/mL challenged by phage population at 1 × 10^7^ PFU/mL. (**G**) T7-resistant cells (*E. coli* R9-5256) at 1 × 10^6^ CFU/mL challenged by phage population at 1 × 10^7^ PFU/mL.

Turbid zones of clearing were observed in all the mixed populations challenged with WT-T7, demonstrating the single-hurdle treatment’s limited ability to control growth in populations containing a subset of resistant cells (Fig. 5). For the mixed populations consisting of 10% and 50% resistant cells, the lawn density of the sensitive sub-population (1 × 10^5^ vs 1 × 10^6^ CFU/ml) influenced the inhibitory impact of the colicin–phage treatments (T7-E1, T7-M, and T7-E1 + T7 M cocktail).

For the mixed populations challenged with the colicin–phage treatments (T7-E1, T7-M, and T7-E1 + T7 M cocktail), the initial lawn density of the sensitive sub-population (1 × 10^5^ vs. 1 × 10^6^ CFU/mL) determined whether we saw localized, turbid zones of clearing versus clear and confluent plaques (as defined as zones of clearing that have merged, resembling a singular large plaque) (37). In the 10% resistant-mixed population, which was comprised of a T7-sensitive subpopulation of 1 × 10^6^ CFU/mL and a T7-resistant subpopulation of 1 × 10^5^ CFU/mL, the double- and triple-hurdle treatments formed confluent and clear plaques, with the colicin–phage cocktail forming the largest zone of clearing (Fig. 5E).

When resistance was increased to 50% of the mixed population, the double- and triple-hurdled treatments formed clear and confluent plaques when the initial lawn density of the mixed population was 1 × 10^6^ CFU/mL (Fig. 5D). When the 50% resistant-mixed population’s initial lawn density was reduced to 1 × 10^5^ CFU/mL, the colicin–phage treatments formed smaller, localized zones of clearings with notably greater turbidity (Fig. 5C). This discrepancy highlights the relationship between bacterial lawn density and phage inhibition in spatially structured environments.

Spatial refuge, defined as heterogeneity in the physical environment, plays a crucial role in phage predation under spatially structured conditions (38). At lower lawn densities, the likelihood of the phage encountering a sensitive host diminishes, hindering phage diffusion and colicin production. Additionally, the presence of resistant cells within the mixed population can further contain the spread of phage diffusion due to a phenomenon known as "numerical refuge" (38, 39)." Numerical refuge occurs in bacterial populations with heterogeneous susceptibilities, where resistant cells act as a physical barrier, preventing phages from encountering susceptible hosts (38). Specific to this work, these refuges influence the underlying dynamical processes of phage–bacteria interactions by creating spatial and susceptibility-based barriers to infection. Spatial refuge directly affects the distribution and movement of phages to reach susceptible hosts, while numerical refuge modulates the population structure and numerical availability of susceptible hosts for infection. Although spatial refuge and numerical refuge are distinct phenomena, both limit phage diffusion through a population density-dependent response. The influence of these dual factors is evident when comparing the 50% mixed populations of lower and higher lawn densities.

When the initial bacterial lawn density was lower, bacterial re-growth was observed between localized zones of clearing, which were smaller in size and notably more turbid (Fig. 5C), whereas at a higher initial lawn density, we found the colicin–phage treatments were able to form clear, confluent plaques of a notably larger size (Fig. 5D) (33).

Our findings contrast with the work of Dennehy et al., who reported an inverse relationship between plaque formation and lawn densities: at higher initial bacterial lawn populations, they observed a decrease in plaque size (24). These diverging findings could be explained by differences in the range of lawn densities investigated between the two studies. We investigated a notably lower range of initial lawn densities (1 × 10^5^ to 1 × 10^6^ CFU/mL) than Dennehy et al. (4 × 10^8^ to 8 × 10^9^ CFU/mL). These differences in lawn density ranges and plaque formation suggest that there is an optimal density between the ranges examined in both studies. At lawn densities above a certain threshold (i.e., plating lawns at an initial concentration approaching stationary phase concentrations), the phage diffusion rate decreases because stationary-phase cells typically exhibit reduced susceptibility to phage predation (24). This is attributed to a decline in the plaque’s wavefront velocity and growth (24). This was the case with Dennehy et al., who reported that plaque formation was negatively impacted when the initial lawn population approached stationary-phase concentrations (24)

Conversely, when lawn density is too low, phages may not encounter enough susceptible hosts to form plaques efficiently, making phage predation the limiting factor (24). This could explain the smaller localized zones of clearing observed in our 1 × 10^5^ CFU/mL populations. Another important factor to consider in the structured environment is the formation of microcolonies within a bacterial lawn. The size of the microcolony is influenced by the initial lawn density, with lower densities associated with a larger colony size. Larger microcolonies have fewer surface bacteria with access to oxygen and nutrients, reducing accessible hosts competent for phage predation. In our case, this would reduce the number of infected cells and subsequently hinder colicin co-expression, adding an additional layer to the smaller and turbid zones of clearing observed in the 1 × 10^5^ CFU/mL populations (24).

Collectively, this demonstrates the population-level factors (i.e., numerical/spatial refuge, stationary phase concentration, and microcolony size) that can impact phage inhibition in spatially structured environments, highlighting the importance of evaluating phage-based antimicrobials under a range of bacterial lawn densities.

#### Unstructured environment challenge matrix

**Fig 6:**
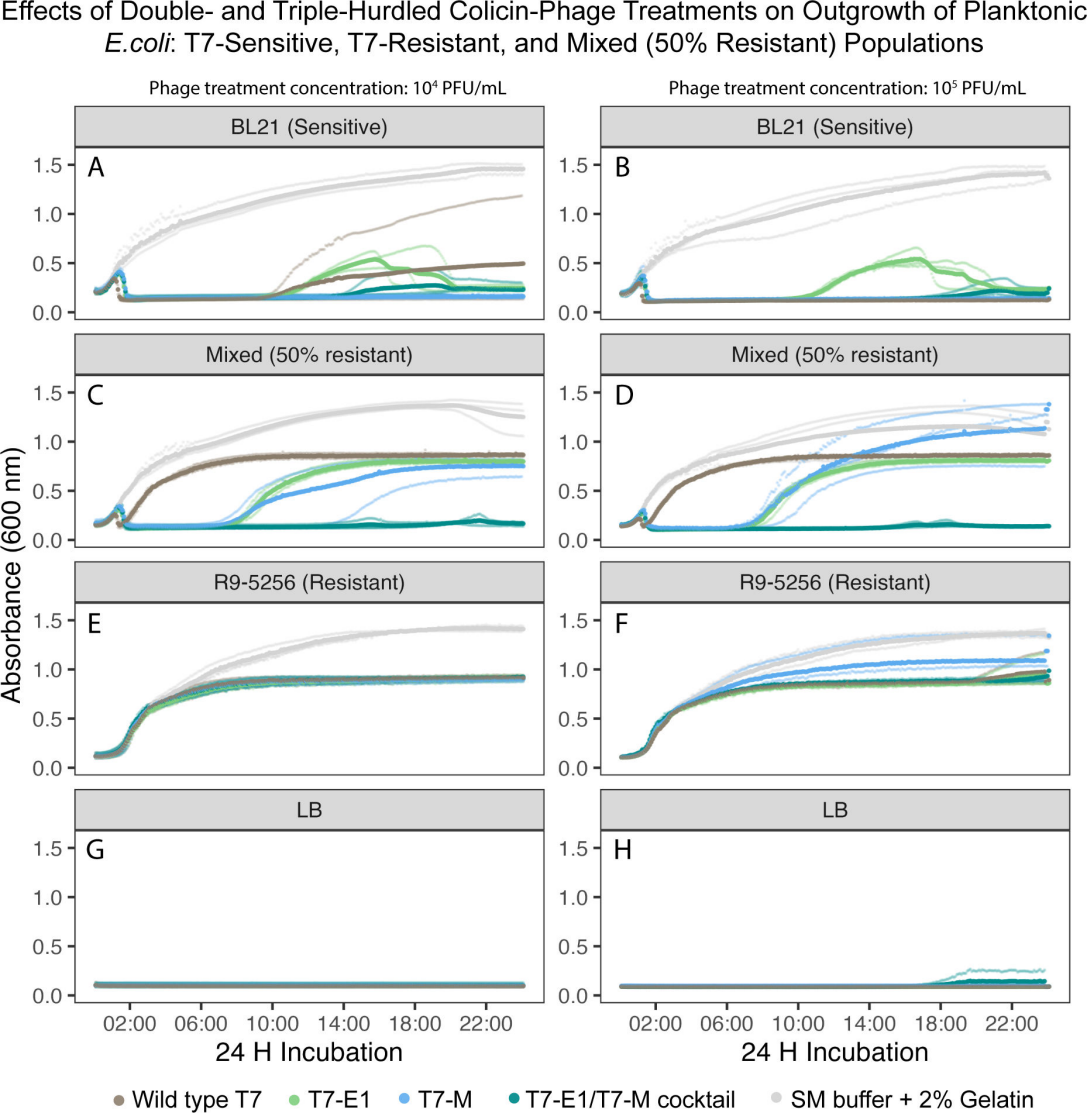
Planktonic challenge matrix growth curves containing the 50% resistant-mixed population challenged with T7-WT, T7-E1, T7-M, T7-E1/T7-M cocktail (1:1 vol/vol), or SM buffer +2% gelatin. (**A**) T7-sensitive cells (*E. coli* BL21) at 1 × 10^7^ CFU/mL challenged by phage population at 1 × 10^4^ PFU/mL. (**B**) T7-sensitive cells (*E. coli* BL21) at 1 × 10^7^ CFU/mL challenged by phage population at 1 × 10^5^ PFU/mL. (**C**) 50% T7-resistant-mixed population (1:1 vol/vol *E. coli* BL21 and *E. coli* R9-5256) at 1 × 10^7^ CFU/mL challenged by phage population at 1 × 10^4^ PFU/mL. (**D**) 50% T7-resistant-mixed population (1:1 vol/vol *E. coli* BL21 and *E. coli* R9-5256) at 1 × 10^7^ CFU/mL challenged by phage population at 1 × 10^5^ PFU/mL. (**E**) T7-resistant cells (*E. coli* R9-5256) at 1 × 10^7^ CFU/mL challenged by phage population at 1 × 10^4^ PFU/mL. (**F**) T7-resistant cells (*E. coli* R9-5256) at 1 × 10^7^ CFU/mL challenged by phage population at 1 × 10^5^ PFU/mL. (**G**) LB negative control challenged by phage population at 1 × 10^4^ PFU/mL. (**H**) LB negative control challenged by phage population at 1 × 10^5^ PFU/mL.

**Fig 7:**
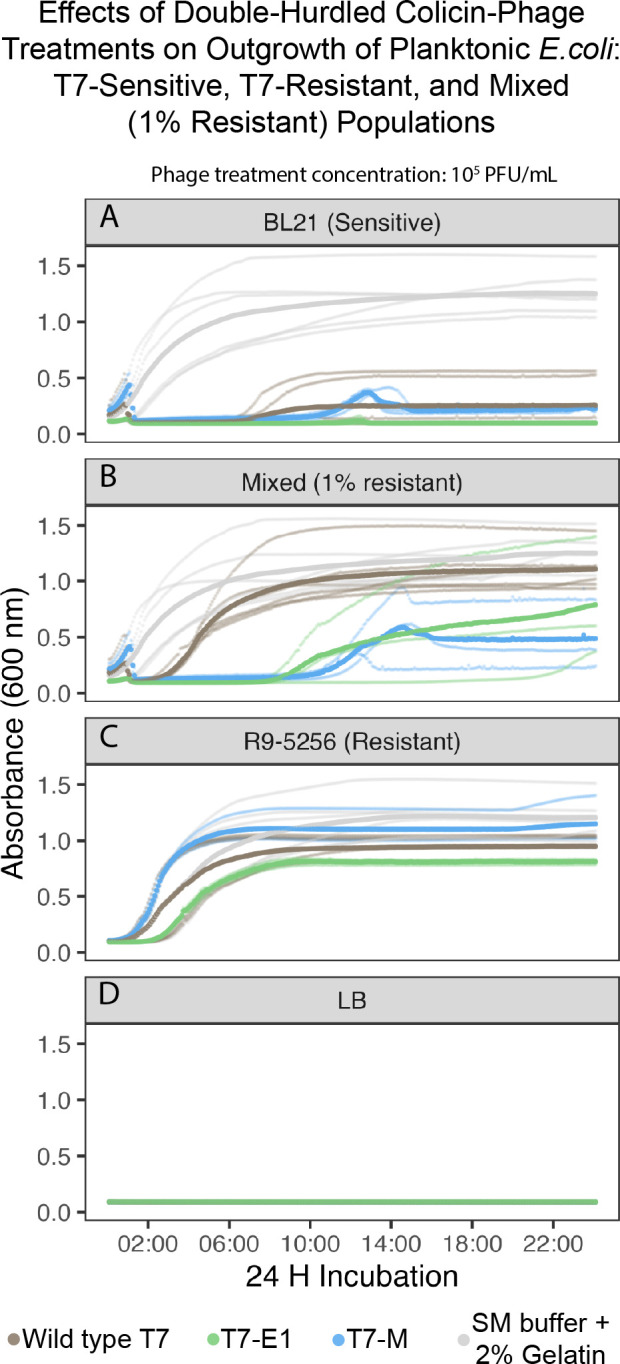
Planktonic challenge matrix growth curves containing the 1% resistant-mixed population challenged with T7-WT, T7-E1, T7-M, or SM buffer +2% gelatin. (**A**) T7-sensitive cells (*E. coli* BL21) at 1 × 10^7^ CFU/mL challenged by phage population at 1 × 10^5^ PFU/mL. (**B**) 1% T7-resistant-mixed population (*E. coli* BL21 at 1 × 10^7^ CFU/mL and *E. coli* R9-5256 at 1 × 10^5^ CFU/mL) challenged by phage population at 1 × 10^5^ PFU/mL. (**C**) T7-resistant cells (*E. coli* R9-5256) at 1 × 10^7^ CFU/mL challenged by phage population at 1 × 10^5^ PFU/mL. (**D**) LB negative control challenged by phage population at 1 × 10^5^ PFU/mL.

**Fig 8:**
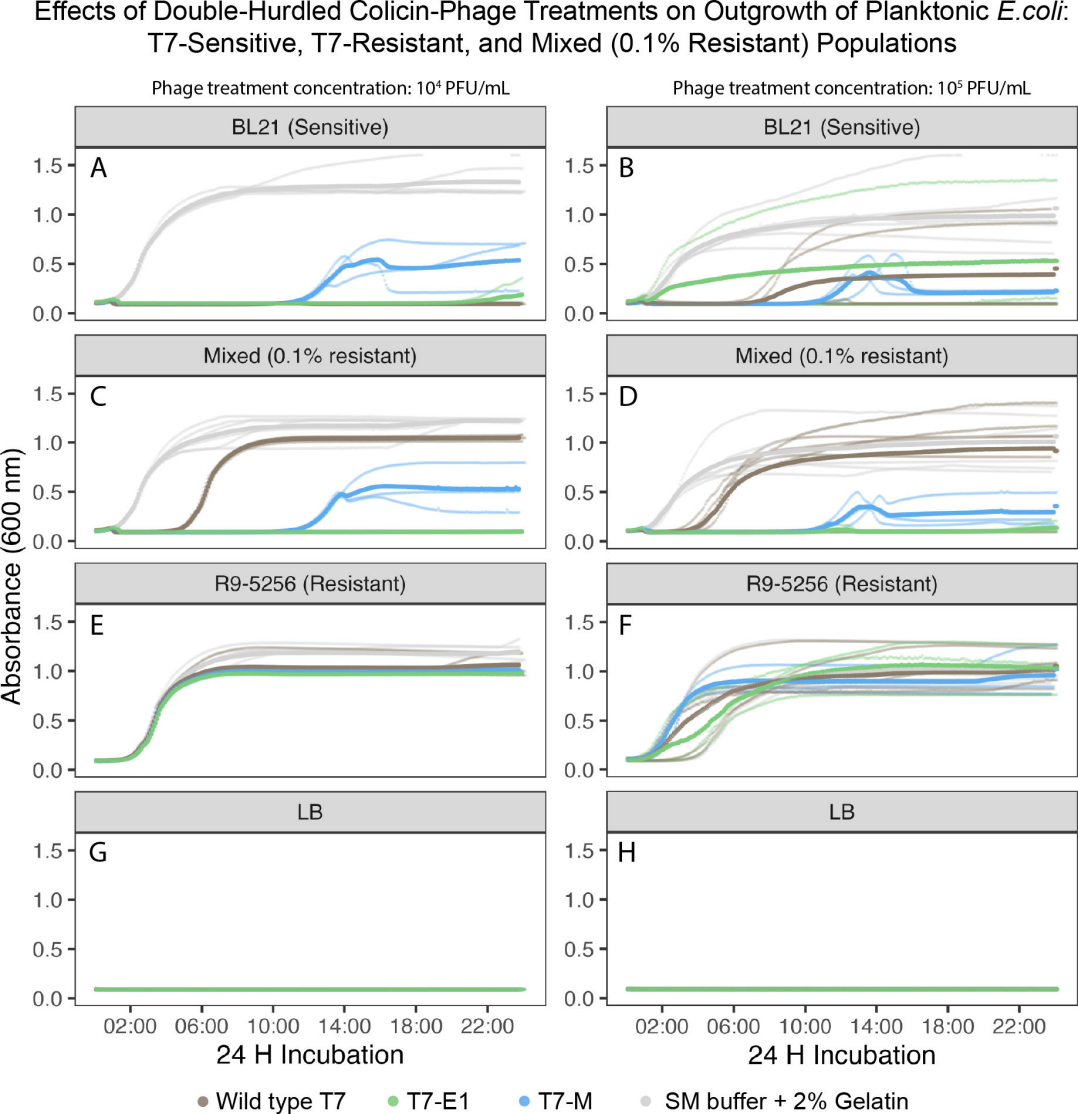
Planktonic challenge matrix growth curves containing the 0.1% resistant-mixed population challenged with T7-WT, T7-E1, T7-M, T7-E1/T7-M cocktail (1:1 vol/vol), or SM buffer +2% gelatin. (**A**) T7-sensitive cells (*E. coli* BL21) at 1 × 10^7^ CFU/mL challenged by phage population at 1 × 10^4^ PFU/mL. (**B**) T7 sensitive cells (*E. coli* BL21) at 1 × 10^7^ CFU/mL challenged by phage population at 1 × 10^5^ PFU/mL. (**C**) 0.1% T7-resistant-mixed population (*E. coli* BL21 at 1 × 10^7^ CFU/mL and *E. coli* R9-5256 at 1 × 10^4^ CFU/mL) challenged by phage population at 1 × 10^4^ PFU/mL. (**D**) 0.1% T7-resistant-mixed population (*E. coli* BL21 at 1 × 10^7^ CFU/mL and *E. coli* R9-5256 at 1 × 10^4^ CFU/mL) challenged by phage population at 1 × 10^5^ PFU/mL. (**E**) T7-resistant cells (*E. coli* R9-5256) at 1 × 10^7^ CFU/mL challenged by phage population at 1 × 10^4^ PFU/mL. (**F**) T7-resistant cells (*E. coli* R9-5256) at 1 × 10^7^ CFU/mL challenged by phage population at 1 × 10^5^ PFU/mL. (**G**) LB negative control challenged by phage population at 1 × 10^4^ PFU/mL. (**H**) LB negative control challenged by phage population at 1 × 10^5^ PFU/mL.

Under planktonic conditions (i.e., well-mixed liquids), the likeliness of the colicin–phage encountering a host is increased compared to structured environments (24). Using liquid media as a simplified model for planktonic conditions, we evaluated the impact of multi-hurdled phage treatments against bacterial populations of varying T7-susceptibilities (Table 2) by measuring their optical density (OD_600_). The growth curves of the untreated sensitive *E. coli* BL21 and resistant *E. coli* R9-5256 populations had similar growth rates at an initial inoculum of 1 × 10^7^ CFU/mL, indicating equal fitness of the two strains used in this study (Fig. 6E and F, 7C, 8E and F).

Overall, the colicin–phage treatments were more effective at inhibiting outgrowth of the mixed population compared to WT-T7 treatment (Fig. 6 to 8). The ratio of resistant cells (0.1%, 1%, or 50%) and the number of hurdles of the treatment (single-WT, double- T7-M or T7-E1, triple—T7-E1/T7-M cocktail) determined if the mixed population’s outgrowth was delayed or completely suppressed.

The triple-hurdle colicin–phage cocktail fully suppressed the outgrowth of a mixed population containing high levels of phage resistance. At viral concentrations of 1 × 10^4^ and 1 × 10^5^ PFU/mL, the colicin–phage cocktail completely suppressed the 50% resistant-mixed population within 2 h of co-inoculation, resulting in a curve resembling the sensitive control population (Fig. 6C and D). At 1 × 10^4^ and 1 × 10^5^ PFU/mL, the double-hurdled treatments (T7-E1 and T7-M) suppressed outgrowth of the mixed population for approximately 7 h on average following co-inoculation (Fig. 6C). Compared to the single-hurdle treatment, the colicin–phages on average delayed outgrowth of the mixed population 5 h longer than what was observed with the mixed population challenged with WT-T7, demonstrating the enhanced inhibitory effect of the colicin hurdle (Fig. 6D).

It is noteworthy that one of the biological replicates of T7-M (1 × 10^4^ PFU/mL) suppressed outgrowth for 14 h following co-inoculation, highlighting the variability in time it can control for growth of mixed population of high resistance (Fig. 6C). The double-hurdle treatments' failure to completely suppress outgrowth suggests that colicin co-expression by the T7-sensitive sub-population failed to surpass the minimum inhibitory concentration (MIC) required for inactivation of the 50% resistant-mixed population.

Overall, the double-hurdle treatments (T7-M and T7-E1) were more effective in inhibiting mixed populations comprised of a smaller sub-population of resistant cells. T7-M (1 × 10^5^ PFU/mL) delayed the outgrowth of the 1% resistant-mixed population by an average of 10 hfollowing co-inoculation (Fig. 7B), which was 4 h longer than what was observed in the 50% resistant-mixed population challenged with T7-M (Fig. 6D). Similarly, T7-E1 delayed outgrowth of the 1% resistant-mixed population by an average of 8 h following co-inoculation (Fig. 7B), which was 1 h longer than what was observed in the 50% resistant-mixed population challenged with T7-E1 (Fig. 6D).

When compared to the growth curve of the 1% resistant-mixed population challenged with WT-T7, the double-hurdle treatments delayed outgrowth for a longer period and yielded a lower final OD_600_ (Fig. 7B). In the 1% resistant-mixed population, outgrowth occurred 2 h following co-inoculation with WT-T7 and reached stationary phase after 8 h, plateauing at a final OD_600_ of 1 nm (Fig. 7B). The growth curve associated with T7-M was biphasic and plateaued after 14 h of coincubation, yielding a final OD_600_ of 0.5 nm (Fig. 7B). For T7-E1, the curve had a reduced slope relative to the untreated (SM buffer) control, indicating a reduction in growth rate and a final OD_600_ below 1 nm (Fig. 7B). The lower final OD_600_ values observed with T7-M and T7-E1 indicate a reduced bacterial load, which is associated with better clinical outcomes. This suggests that the double-hurdle treatments can achieve more effective bacterial suppression than traditional single-hurdle treatments. There was some variation within biological replicates of the double-hurdle treatments, especially within T7-E1’s biological replicates, where the timepoint in which we observed outgrowth ranged from 6 to 22 h post-inoculation. Notably, one of T7-M’s biological replicates completely suppressed the resistant-mixed population. At 1 × 10^4^ PFU/mL, the double-hurdled treatments (T7-E1 and T7-M) could not suppress or delay outgrowth (data not shown).

When the mixed population’s resistant sub-population was reduced to 0.1%, the inhibitory effect of double-hurdle treatments improved. At both concentrations tested (1 × 10^4^ and 1 × 10^5^ PFU/mL), T7-E1 fully suppressed the outgrowth of the 0.1% resistant-mixed population across the three biological replicates (Fig. 8C and D). These results suggest sufficient phage-host infections are occurring, enabling the co-expression of colicin-E1 to surpass the MIC of the 0.1% resistant-mixed population. T7-M did not fully suppress the 0.1% resistant-mixed population, but its delayed effect on outgrowth improved relative to the mixed populations with a higher prevalence of resistant cells (1% and 50%). On average, T7-M delayed outgrowth by 12 h after co-inoculation at 1 × 10^4^ and 1 × 10^5^ PFU/mL (Fig. 8C and D). T7-M delayed outgrowth over a longer period and yielded a lower final OD value than the single-hurdle treatment (Fig. 8C and D). When challenged with WT-T7 (1 × 10^4^ and 1 × 10^5^ PFU/mL), outgrowth of the 0.1% resistant-mixed population occurred 2 h following co-inoculation and reached stationary phase within 10 h, plateauing at a final OD_600_ of 1 nm (Fig. 8C and D). For comparison, the growth curve associated with T7-M (1 × 10^4^ PFU/mL) plateaued at a final OD_600_ of ~0.5 after 14 h (Fig. 8C). It is worth noting that T7-M fully suppressed outgrowth in two of the three biological replicates at the concentration of 1 × 10^5^ PFU/mL (Fig. 8D). In the replicate with outgrowth, the curve plateaued at the reduced OD_600_ value of 0.5 nm. This variability indicates the potential for T7-M to fully suppress growth at higher concentrations, suggesting that higher or more precise phage concentrations could achieve more consistent results across all replicates.

The overall lower OD_600_ observed with the population challenged with T7-M (compared to WT-T7) indicates a more substantial reduction in bacterial growth, demonstrating T7-M’s enhanced efficacy in controlling and reducing the population of resistant bacteria. Furthermore, the longer delayed effect of T7-M compared to the WT-T7 is significant as it suggests a prolonged suppression of bacterial regrowth, providing a larger window before additional treatment may be necessary, which is crucial for effective infection management and resistance prevention strategies.

It appears numerical refuge also impacts the efficacy of the double treatments under planktonic conditions. This is evident when comparing the inhibitory effect of the double-hurdle treatments across mixed populations with different resistant-to-sensitive cell ratios. When the relative resistant population was low (0.1% or 1%), the double-hurdled treatments (T7-E1 and T7-M) were more effective in controlling growth of the mixed population. However, when resistance was increased to 50%, the double-hurdle treatments were less effective in controlling outgrowth. This suggests that the higher prevalence of resistant cells in the 50% mixed population offers a numerical refuge by diluting the likelihood of the colicin–phage physically encountering a sensitive host (38). As such, the double-hurdle treatments may eliminate all the locally accessible sensitive hosts before they can be replenished by cell division, thus limiting the spread of the infection and halting colicin co-expression (38). Interestingly, the triple-hurdle treatment (T7-E1/T7-M cocktail) appears unaffected by numerical refuge, as demonstrated by the complete suppression of the 50% mixed populations. This suggests that the three hurdles' compounding inhibitory effect could counteract the resistant sub-population’s spatial limitations. Specifically, the lysis of the resistant cells by the colicins could enable the phage to function more freely within the planktonic cultures, enhancing its likelihood of encountering a sensitive host.

Collectively, these results highlight a synergism between the colicin hurdles and treatment efficacy. As the prevalence of T7-resistance increases in a heterogeneous population, more hurdles are required to completely suppress outgrowth. This relationship does not appear linear, as demonstrated by the effectiveness of the triple-hurdle treatment in the 50% resistant-mixed population. Complete suppression by the double-hurdled treatments is only consistently achieved when the resistant sub-population is reduced 500-fold to 0.1%. This suggests a dynamic relationship between the concentration of the antimicrobial compounds of the double-hurdle treatments and the prevalence of resistant sub-populations, indicating the influence of numerical refuge.

Overall, these results indicate that the double-hurdle colicin–phage treatment would work well in planktonic communities where a T7-phage pressure has not been applied, as we would expect low levels of pre-existing T7-phage resistance. When phage-resistant cells constitute a higher proportion of the population (>1%), the addition of a third hurdle, like the colicin–phage cocktail, is likely needed to achieve a microbiological successful outcome. Future work investigating the colicin concentration achieved as a factor of sensitive cells is warranted to reduce variability and optimize the double-hurdle treatment efficacy.

### Exploring *de novo* resistance to multiple-hurdle in phage-sensitive populations

Unexpected outgrowth of the sensitive control population (i.e., wells containing 0% resistant cells) challenged with the double-hurdle and WT-T7 treatments was occasionally observed in a sub-set of biological replicates in the planktonic challenge matrix. The meticulous implementation of aseptic techniques excludes contamination as a plausible cause of the observed outgrowth. This suggests that the observed phenomenon is more likely attributed to a form of resistance within the sensitive population.

Attempts to generate genetically resistant mutants for downstream characterization failed, yielding isogenic populations that regained sensitivity to the phage treatments during subsequent confirmatory spot tests.

Previous studies have raised concern regarding the loss of transgenic proteins in synthetically engineered phages due to the tragedy of the commons, in which beneficial exogenous genes are selected against and are evolutionary unstable (16). Samples of the colicin–phages following co-inoculation with the isogenic populations underwent PCR, which confirmed the presence of the colicin gene. After eliminating the possibility that temporary sensitivity was due to a loss of colicin co-expression, we adjusted parameters of the fluctuation assays (i.e., phage concentration, culture volume, incubation period, medium) known to increase the number of mutants per culture (40). Despite these optimizations, our assays only produced transient resistance, suggesting that the predominant mechanism selected for by phage treatments is a form of non-canonical resistance arising from population-wide phenotypic heterogeneity of the sensitive population (41).

Antimicrobial persistence is an example of phenotypic heterogeneity that yields a transiently tolerant phenotype (42). Persistence manifests as a dormant subset of cells within a predominantly sensitive isogenic population (42). The increased resiliency of persister cells is attributed to their growth-arrested state arising from fluctuations in gene expression (42). Environmental stress, like nutrient unavailability or antimicrobial activity, is associated with persister formation (42). Given the relationship between persistence and stress, we explored the impact of the double-hurdle treatments on persistence frequency using a modified fluctuation assay (27).

**Fig 9:**
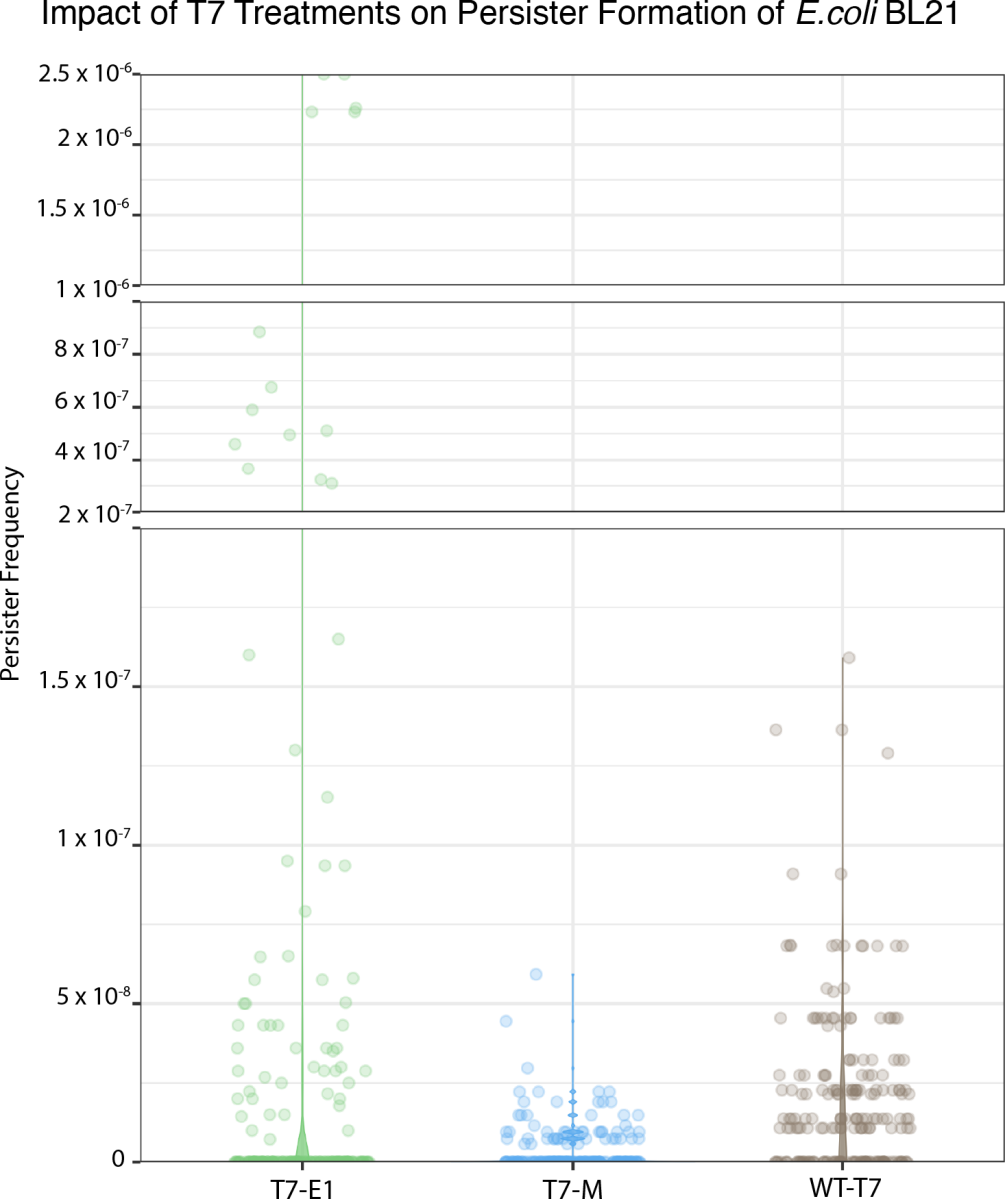
Persister frequency resulting from phage treatment at 1 × 10^8^ PFU/mL. Isogenic populations were seeded with T7-sensitive *E. coli* BL21 at 1 × 10^2^ CFU/mL and exposed to the phage treatment after 20 h. The parallel cultures were re-exposed to the phage treatment at 1 × 10^8^ PFU/mL prior to plating on over-dried plates. After 72 h of incubation, persister colony-forming units were counted, and all persister populations were confirmed transiently tolerant in subsequent spot assays. Circles represent technical replicas.

Relative to WT-T7, there was a notable difference in persister frequency resulting from the double-hurdle treatment (Fig. 9). T7-WT significantly increased the likelihood of persister formation by 2.4-fold compared to T7-M (*P* < 0.005). In contrast, T7-E1 demonstrated a 12-fold increase relative to WT-T7, indicating a significantly higher propensity to induce persister formation (*P* < 0.0001). The disparity in persister frequency may be attributed to the distinct modes of action exhibited by colicin E1 and colicin M. The compounding stress of colicin E1’s pore-forming mechanism and the phage lysogen could elicit a more pronounced stress response compared to colicin M’s less direct mode of action, which inhibits peptidoglycan synthesis by blocking regeneration of the bactoprenyl-P carrier lipid (19, 20).

Notably, our findings differed from a study by Jin X et al., which reported that colicin E1 eradicated persister formation in rifampin pre-treated *E. coli* K361 (43). However, key distinctions in our experimental design could account for these divergent results. First, the antimicrobial combinations were different: while Jin X et al. used rifampin and colicin E1, we employed phage and colicin E1. Additionally, Jin X et al. examined sequential antimicrobial treatments, whereas we investigated simultaneous exposure. Finally, our observation period significantly exceeded theirs (72 h compared to 3 h), potentially enabling us to capture slower-growing persisters.

Regardless, this work contributes to our understanding of antimicrobial persistence, which is intricate and highly dynamic in nature. The absence of a definitive mechanism behind persister formation further supports this observation. Although various genetic factors have historically been linked to persistence, no single gene has been demonstrated to cause persistence (44). Instead, persistence is believed to be the product of disruptions in cellular networks arising from environmental stress (27, 45). As such, various genetic pathways could induce persistence if the stress on cellular networks surpassed a certain threshold of disruption, thus explaining the conditional element observed with T7-E1 compared to those of T7-M and Jin et al. (43, 45).

Together, T7-M and T7-E1 illustrate the versatility of stress-induced persistence. T7-M adheres to the logic that more antimicrobials lead to better outcomes, while T7-E1 challenges this notion. Future work investigating gene expression differences between the double-hurdle treatments is warranted to understand how these mechanisms yield different persistence outcomes from a transcriptomic lens.

### Conclusion

Collectively, this work addresses the following two dimensions of AMR: the presence of resistance present prior to treatment and the emergence of non-canonical resistance through population heterogeneity following treatment. The varying efficacy of the multi-hurdle treatments between the challenge matrix and the fluctuation assay demonstrates the intricate modalities of AMR. This underscores the need to tailor therapies not just to target specific pathogens but to specific manifestations of resistance (e.g., genetic resistance within a mixed bacterial population vs non-canonical resistance within a sensitive population).

Moreover, the interplay between selective pressures exerted by the antimicrobial and bacterial stress response should be assessed to determine its propensity for inducing persistence, particularly for combinational treatments. This requires the development of rapid diagnostic platforms for non-canonical resistance, as the MIC-based approaches are insufficient at distinguishing between sensitive and antimicrobial-tolerant cultures (42). Enhanced diagnostics, coupled with novel therapeutics targeting non-canonical resistance, will diminish the occurrence of treatment failure stemming from population heterogeneity.

Given non-canonical resistance’s role in the progressive evolution of genetic resistance, targeting non-canonical resistance could be a more strategic and pre-emptive solution to interrupt the development and spread of AMR (41). Ultimately, adopting a more holistic framework allows for more effective approaches to combat the dynamic nature of bacterial populations, ensuring sustainable antimicrobial therapies for future generations.

## SUPPLEMENTAL MATERIAL

Supplemental material spectrum.00793-24-s0001.pdf Contains construct DNA and sequences for colicin genes as well as colicin western blot.

ASM does not own the copyrights to Supplemental Material that may be linked to, or accessed through, an article. The authors have granted ASM a non-exclusive, world-wide license to publish the Supplemental Material files. Please contact the corresponding author directly for reuse.
