# Supplementary material for: Assessment of resistance to colicinogenic synthetic phage antimicrobial system
Source: Microbiol Spectr. 2024 Oct 15;12(11):e00793-24. doi: 10.1128/spectrum.00793-24 (PMC11537092; doi:10.1128/spectrum.00793-24)
Supplement: Supplemental material — Contains construct DNA and sequences for colicin genes as well as colicin western blot. [file spectrum.00793-24-s0001.pdf]

1. Supplementary information

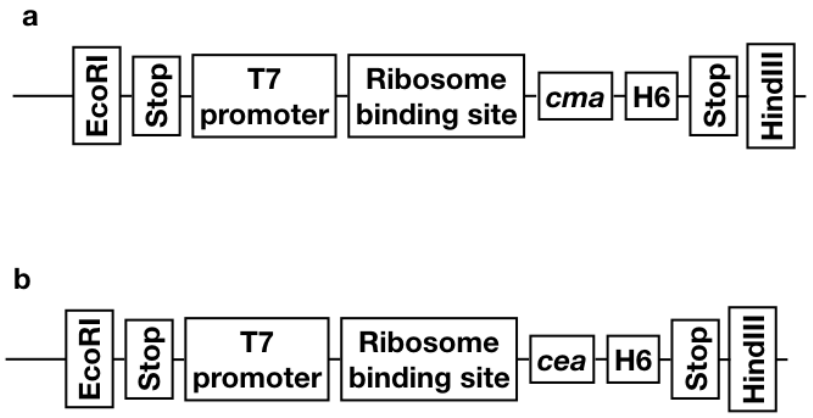

Supplemental Figure 1. Diagram of DNA constructs. (a) Our construct containing *cma*. (b) Our construct containing *cea*.

GAATTC[1]TGAATGAATGA[2]TAATTAATACGACTCACTATAG[3]GGAGACCACAAC
GGTTTCCCTCTAGAAATAATTTTGTTTAACTTTAAGAAGGAG[4]ATATACATAT
G[5]GAAACCGCGGTGGCGTACTATAAGGACGGCGTTCCGTACGACGATAAGGG
TCAGGTGATCATTACCCTGCTGAACGGTACCCCGGATGGTAGCGGTAGCGGT
GGCGGTGGCGGTAAGGGCGGTAGCAAAAGCGAGAGCAGCGCGGCGATTCAT
GCGACCGCGAAGTGGAGCACCGCGCAACTGAAGAAAACCCAGGCGGAACAA
GCGGCGCGTGCGAAAGCGGCGGCGGAGGCGCAGGCGAAAGCGAAAGCGAA
CCGTGACGCGCTGACCCAACGTCTGAAGGATATTGTTAACGAAGCGCTGCGT
CACAACGCGAGCCGTACCCCGAGCGCGACCGAGCTGGCGCATGCGAACAAC
GCGGCGATGCAGGCGGAGGACGAACGTCTGCGTCTGGCGAAGGCGGAGGAA
AAGGCGCGTAAAGAGGCGGAGGCGGCGGAAAAAGCGTTCCAGGAAGCGGAA
CAACGTCGTAAGGAGATCGAACGTGAGAAAGCGGAAACCGAGCGTCAACTG
AAGCTGGCGGAAGCGGAGGAAAAACGTCTGGCGGCGCTGAGCGAGGAAGCG

AAAGCGGTGGAGATTGCGCAGAAGAAACTGAGCGCGGCGCAAAGCGAAGTG
GTTAAGATGGACGGCGAGATCAAAACCCTGAACAGCCGTCTGAGCAGCAGC
ATTCACGCGCGTGATGCGGAAATGAAGACCCTGGCGGGTAAACGTAACGAGC
TGGCGCAGGCGAGCGCGAAGTACAAAGAACTGGACGAGCTGGTTAAGAAAC
TGAGCCCGCGTGCGAACGATCCGCTGCAAAACCGTCCGTTCTTTGAAGCGAC
CCGTCGTCGTGTGGGTGCGGGCAAGATCCGTGAGGAAAAGCAGAAACAAGTT
ACCGCGAGCGAGACCCGTATCAACCGTATTAACGCGGACATCACCCAGATTC
AAAAAGCGATCAGCCAAGTGAGCAACAACCGTAACGCGGGCATTGCGCGTG
TTCACGAAGCGGAGGAAAACCTGAAGAAAGCGCAGAACAACCTGCTGAACA
GCCAAATCAAGGACGCGGTGGATGCGACCGTTAGCTTCTATCAGACCCTGAC
CGAAAAATACGGCGAGAAGTATAGCAAAATGGCGCAAGAGCTGGCGGACAA
GAGCAAAGGCAAGAAAATTGGTAACGTGAACGAAGCGCTGGCGGCGTTTGA
GAAGTACAAAGATGTTCTGAACAAGAAATTCAGCAAGGCGGACCGTGATGC
GATCTTTAACGCGCTGGCGAGCGTGAAGTATGACGATTGGGCGAAACACCTG
GATCAGTTCGCGAAGTACCTGAAAATTACCGGCCACGTTAGCTTTGGTTATGA
CGTGGTTAGCGATATCCTGAAGATTAAAGACACCGGCGATTGGAAACCGCTG
TTCCTGACCCTGGAAAAGAAAGCGGCGGACGCGGGTGTGAGCTACGTGGTTG
CGCTGCTGTTTAGCCTGCTGGCGGGTACCACCCTGGGTATCTGGGGTATCGCG
ATTGTGACCGGTATCCTGTGCAGCTATATTGATAAGAACAAACTGAACACCA
TCAACGAGGTTCTGGGTATTCACCATCACCACCACCAC[6]TAA[7]GTAACTAAAA
GCTT[8]

Supplemental Figure 2. Insert for cea-his. Sequence Length: 1702bp. Sequence Features: 1. *Eco*RI restriction site. 2. Stop codons in all three frames to prevent attachment to capsid protein. 3. T7 promoter. 4. Ribosome Binding Site. 5. Start codon for colicin E1 protein. 6. Histidine tag. 7. Stop codon. 8. *Hind*III restriction site.

GAATTC[1]TGAATGAATGA[2]TAATTAATACGACTCACTATAG[3]GGAGACCACAAC
GGTTTCCCTCTAGAAATAATTTTGTTTAACTTTAAGAAGGAG[4]ATATACATAT
G[5]GAGACCCTGACCGTGCATGCGCCGAGCCCGAGCACCAACCTGCCGAGCTA
TGGTAACGGTGCGTTCAGCCTGAGCGCGCCGCACGTGCCGGGTGCGGGTCCG
CTGCTGGTTCAGGTGGTTTACAGCTTCTTTCAAAGCCCGAACATGTGCCTGCA
GGCGCTGACCCAACTGGAAGATTATATCAAGAAACACGGTGCGAGCAACCC
GCTGACCCTGCAGATCATTAGCACCAACATCGGTTACTTCTGCAACGCGGAC
CGTAACCTGGTGCTGCACCCGGGCATTAGCGTTTACGATGCGTATCACTTTGC
GAAGCCGGCGCCGAGCCAGTACGACTATCGTAGCATGAACATGAAACAAAT
GAGCGGTAACGTGACCACCCCGATCGTTGCGCTGGCGCACTATCTGTGGGGT
AACGGCGCGGAGCGTAGCGTTAACATCGCGAACATTGGCCTGAAGATTAGCC
CGATGAAGATCAACCAAATCAAGGATATCATCAAGAGCGGTGTGGTTGGCAC
CTTCCCGGTTAGCACCAAGTTTACCCACGCGACCGGTGACTACAACGTTATCA
CCGGTGCGTATCTGGGCAACATTACCCTGAAAACCGAAGGCACCCTGACCAT
CAGCGCGAACGGTAGCTGGACCTACAACGGCGTGGTTCGTAGCTACGACGAT
AAGTATGACTTCAACGCGAGCACCCACCGTGGTATCATTGGCGAGAGCCTGA
CCCGTCTGGGTGCGATGTTTAGCGGCAAAGAATATCAGATTCTGCTGCCGGG
CGAGATCCACATTAAGGAAAGCGGCAAACGTCATCACCACCACCACCAC[6]TA
A[7]GTAACTAAAAGCTT[8]

Supplemental Figure 3. Insert for cma-his. Sequence Length: 949bp. Sequence Features: 1. *Eco*RI restriction site. 2. Stop codons in all three frames to prevent attachment to capsid protein. 3. T7 promoter. 4. Ribosome Binding Site. 5. Start codon for colicin M protein. 6. Histidine tag. 7. Stop codon. 8. *Hind*III restriction site.

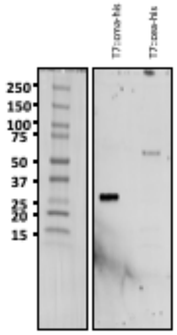

Supplemental Figure 4. The western blot of each of the phage lysates shows the respective sizes of the bacteriocins produced.
